# Refractory Hypotension after Liver Allograft Reperfusion: A Case of Dynamic Left Ventricular Outflow Tract Obstruction

**DOI:** 10.3389/fmed.2016.00003

**Published:** 2016-02-16

**Authors:** Michael Essandoh, Andrew Joseph Otey, Adam Dalia, Elisabeth Dewhirst, Andrew Springer, Mitchell Henry

**Affiliations:** ^1^Department of Anesthesiology, The Ohio State University Wexner Medical Center, Columbus, OH, USA; ^2^Department of Surgery, The Ohio State University Wexner Medical Center, Columbus, OH, USA

**Keywords:** postreperfusion syndrome, dynamic left ventricular outflow tract obstruction, systolic anterior motion of the mitral valve, paradoxical hypotension, transesophageal echocardiography, mitral regurgitation

## Abstract

Hypotension after reperfusion is a common occurrence during liver transplantation following the systemic release of cold, hyperkalemic, and acidic contents of the liver allograft. Moreover, the release of vasoactive metabolites such as inflammatory cytokines and free radicals from the liver and mesentery, compounded by the hepatic uptake of blood, may also cause a decrement in systemic perfusion pressures. Thus, the postreperfusion syndrome (PRS) can materialize if hypotension and fibrinolysis occur concomitantly within 5 min of reperfusion. Treatment of the PRS may require the administration of inotropes, vasopressors, and intravenous fluids to maintain hemodynamic stability. However, the occurrence of the PRS and its treatment with inotropes and calcium chloride may lead to dynamic left ventricular outflow tract obstruction (DLVOTO) precipitating refractory hypotension. Expedient diagnosis of DLVOTO with transesophageal echocardiography is extremely vital in order to avoid potential cardiovascular collapse during this critical period.

## Introduction

Liver allograft reperfusion can be associated with significant cardiovascular instability and may require the administration of inotropes, vasopressors, and intravenous fluids, in order to avert cardiovascular collapse (1–8). The etiology of this cardiovascular depression is multifactorial and can result from several factors after unclamping of the portal vein, including: systemic release of the allograft preservative solution (cold and hyperkalemic fluid), blood-volume uptake by the liver allograft, the release of vasoactive mediators (such as inflammatory cytokines and free radicals), and acidic blood return from the liver and mesentery (1–5, 9, 10). The combination of these factors can result in the postreperfusion syndrome (PRS), a phenomenon initially described by Aggarwal et al. in 1987 (1–4). Significant hypotension may also occur after portal vein unclamping due to several other factors, such as: acute pulmonary embolus (from an inferior vena cava or portal vein thrombus), venous air embolism, acute right ventricular failure, dynamic left ventricular outflow tract obstruction (DLVOTO), and preload limitations (from torsion or obstruction of the inferior vena cava and massive blood loss) (1, 2, 6–8, 11). The etiology of the hypotension therefore has to be investigated in a timely fashion, to ensure rapid and appropriate pharmacological therapy, and optimal patient outcomes.

We present a case of refractory hypotension precipitated by severe DLVOTO after liver reperfusion in a patient with left ventricular basal septal hypertrophy and a long anterior mitral leaflet. We also discuss the utility of transesophageal echocardiography (TEE) as a diagnostic tool, and a guide for hemodynamic management in this dynamic setting.

## Case Report

A 54-year-old male with end-stage liver disease presented emergently for orthotopic liver transplantation (OLT). Written consent was obtained from the patient for publication. The patient’s past medical history was significant for non-alcoholic steatohepatitis cirrhosis, hypertension, refractory ascites (requiring multiple paracenteses), hepatic encephalopathy, and hepatorenal syndrome. The patient had an extensive preoperative workup, which was inclusive of an esophagogastroduodenoscopy (EGD), a transthoracic echocardiogram, and an adenosine technetium-99m sestamibi stress single-photon emission computed tomography (SPECT) myocardial perfusion imaging study. Following the echocardiogram, results revealed hyperdynamic LV function (ejection fraction of 70–75%), normal LV size, normal right ventricular size and function, mild tricuspid insufficiency, absence of intracardiac or transpulmonary shunts, and normal mitral valve (MV) leaflet morphology with trace mitral regurgitation. These normal echocardiographic findings suggested that a nuclear perfusion scan would be adequate to rule out hemodynamically significant obstructive coronary artery disease, as opposed to a dobutamine stress echocardiogram. Therefore, an adenosine stress test was performed 7 months prior to surgery. Results did not reveal any myocardial ischemia or prior myocardial injury, and demonstrated a hyperdynamic LV (ejection fraction of 83%). An EGD performed 2 months previously demonstrated the absence of esophageal variceal disease. Pertinent preoperative laboratory results included an elevated creatinine level of 1.5 mg/dL (normal < 1.3 mg/dL), a low hematocrit level of 21% (normal > 39%), a low platelet count of 99 K/µL (normal > 150 K/µL), an elevated INR of 1.7 (normal < 1.1), and an elevated PTT of 40 s (normal < 34 s).

**Figure 1:**
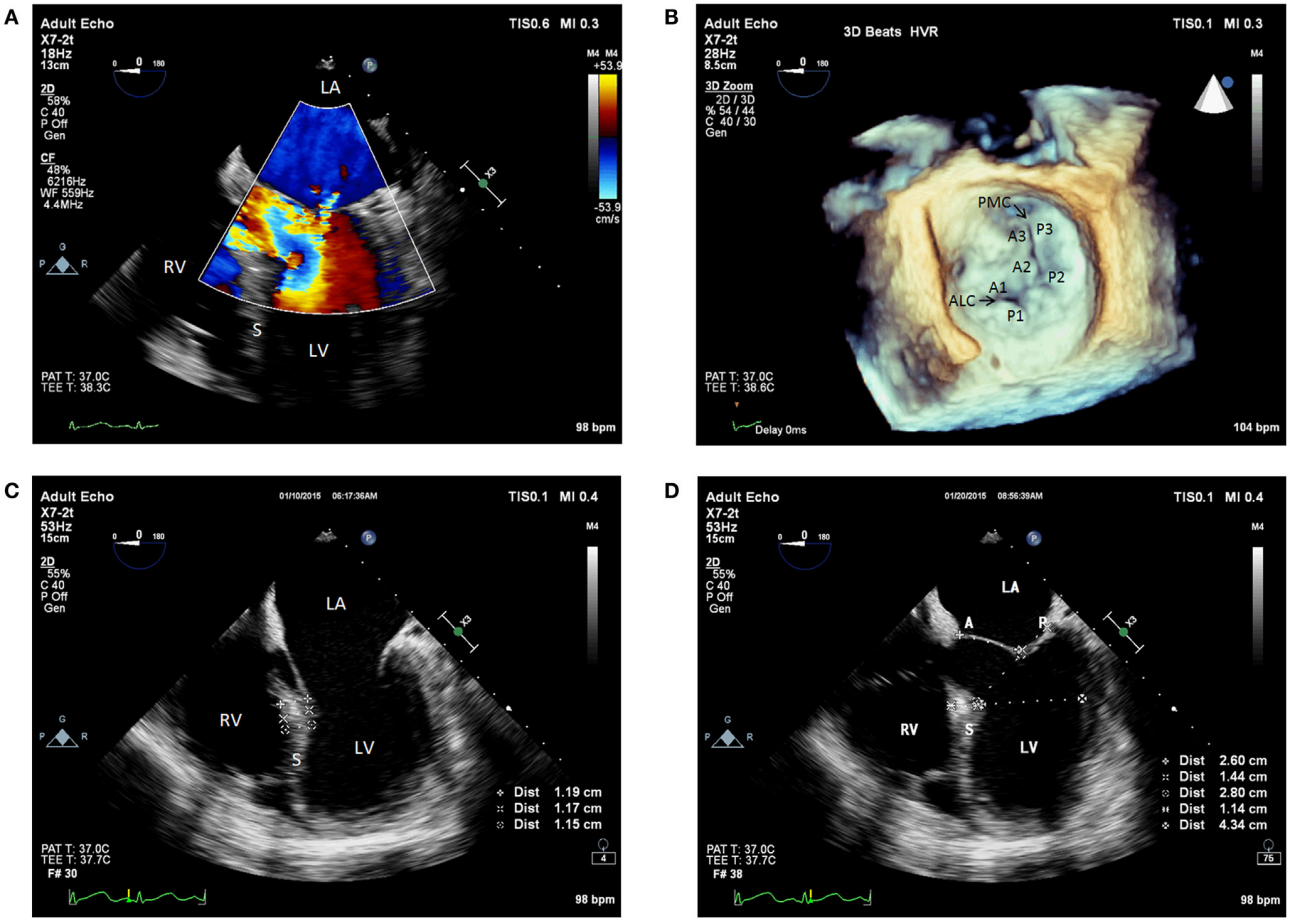
**(A)** Pre-anhepatic 2D TEE midesophageal modified five-chamber view with color flow Doppler showing trace to mild mitral regurgitation. **(B)** Pre-anhepatic 3D TEE en face view of the mitral valve visualized from the left atrial perspective indicating normal coaptation of the mitral leaflets during systole. **(C)** Pre-anhepatic 2D TEE modified five-chamber view in diastole demonstrating mild basal interventricular septal hypertrophy. **(D)** Pre-anhepatic 2D TEE modified five-chamber view in systole demonstrating the absence of risk factors for systolic anterior motion of the mitral valve leaflets. Absent risk factors include normal values for basal LV systolic dimension (4.3 cm), ratio of anterior to posterior mitral leaflet coapted length (1.86), and distance between the mitral coaptation point and the basal LV septum (2.8 cm). LV, left ventricle; RV, right ventricle; LA, left atrium; S, interventricular septum; ALC, anterolateral commissure; PMC, posteromedial commissure; A, anterior mitral valve leaflet; A1, first segment of the anterior mitral valve leaflet; A2, second segment of the anterior mitral valve leaflet; A3, third segment of the anterior mitral valve leaflet; P, posterior mitral valve leaflet; P1, first segment of the posterior mitral valve leaflet; P2, second segment of the posterior mitral valve leaflet; P3, third segment of the posterior mitral valve leaflet.

The patient was taken into the operating room for OLT. The standard ASA monitors were placed, as well as a right radial arterial catheter for hemodynamic monitoring. Rapid-sequence induction of general anesthesia was performed, and intubation of the trachea was completed uneventfully. For hemodynamic monitoring, a right internal jugular venous oximetric pulmonary arterial catheter was inserted. Additionally, an 8.5 Fr rapid infusion catheter was inserted into the right antecubital vein for volume administration. Intraoperative TEE evaluation (X7-2t transducer; Philips Healthcare, Andover, MA, USA) showed a hyperdynamic LV (ejection fraction of 65%), normal right ventricular systolic function, and normal biventricular sizes. The LV basal septum was mildly hypertrophied, measuring 1.2 cm (normal < 1.1 cm). Furthermore, the MV had normal leaflet morphology on 2-dimensional and 3-dimensional assessment, without any evidence of systolic anterior motion (SAM)/DLVOTO. However, the anterior MV leaflet was elongated, measuring 2.6 cm, and the posterior leaflet length was 1.4 cm (Figures 1A–D).

Incision was performed followed by the drainage of 23 L of ascitic fluid. This resulted in systemic hypotension, which was successfully treated with phenylephrine and fluid boluses. A TEE assessment was performed and had similar findings to the baseline examination. Significant blood loss was experienced during the pre-anhepatic phase and required transfusion of packed red blood cells. Point-of-care thromboelastometry was utilized intraoperatively to guide hemostatic therapy. This was achieved using the ROTEM^®^ device (Tem International GmbH, Munich, Germany). A baseline ROTEM revealed deficiencies in the patient’s clotting factors, fibrinogen and platelets, but no evidence of fibrinolysis. To correct the coagulopathy, a combination of fresh frozen plasma, platelets, and cryoprecipitate were administered. Norepinephrine and vasopressin infusions were also initiated to support the patient’s systemic vascular resistance (SVR) in order to maintain adequate systemic perfusion pressures.

The anhepatic phase was associated with worsening hypotension after partial inferior vena cava clamping, which was treated with increased doses of norepinephrine and vasopressin infusions. The liver allograft transplantation was performed using the piggyback technique. After completion of the cavo-caval piggyback anastomosis, the liver was retrogradely flushed, and the portal vein anastomosis was subsequently completed. Retrograde flushing of the allograft was performed to remove the preservative solution and hepatic metabolites before antegrade reperfusion. Immediately prior to reperfusion, the patient had a systemic blood pressure (BP) of 126/73 mmHg, and a heart rate (HR) of 95 beats/min. The patient was prophylactically treated for PRS immediately prior to reperfusion with fluids, 10 mcg of epinephrine, 500 mg of calcium chloride, and 100 mcg of phenylephrine.

**Figure 2:**
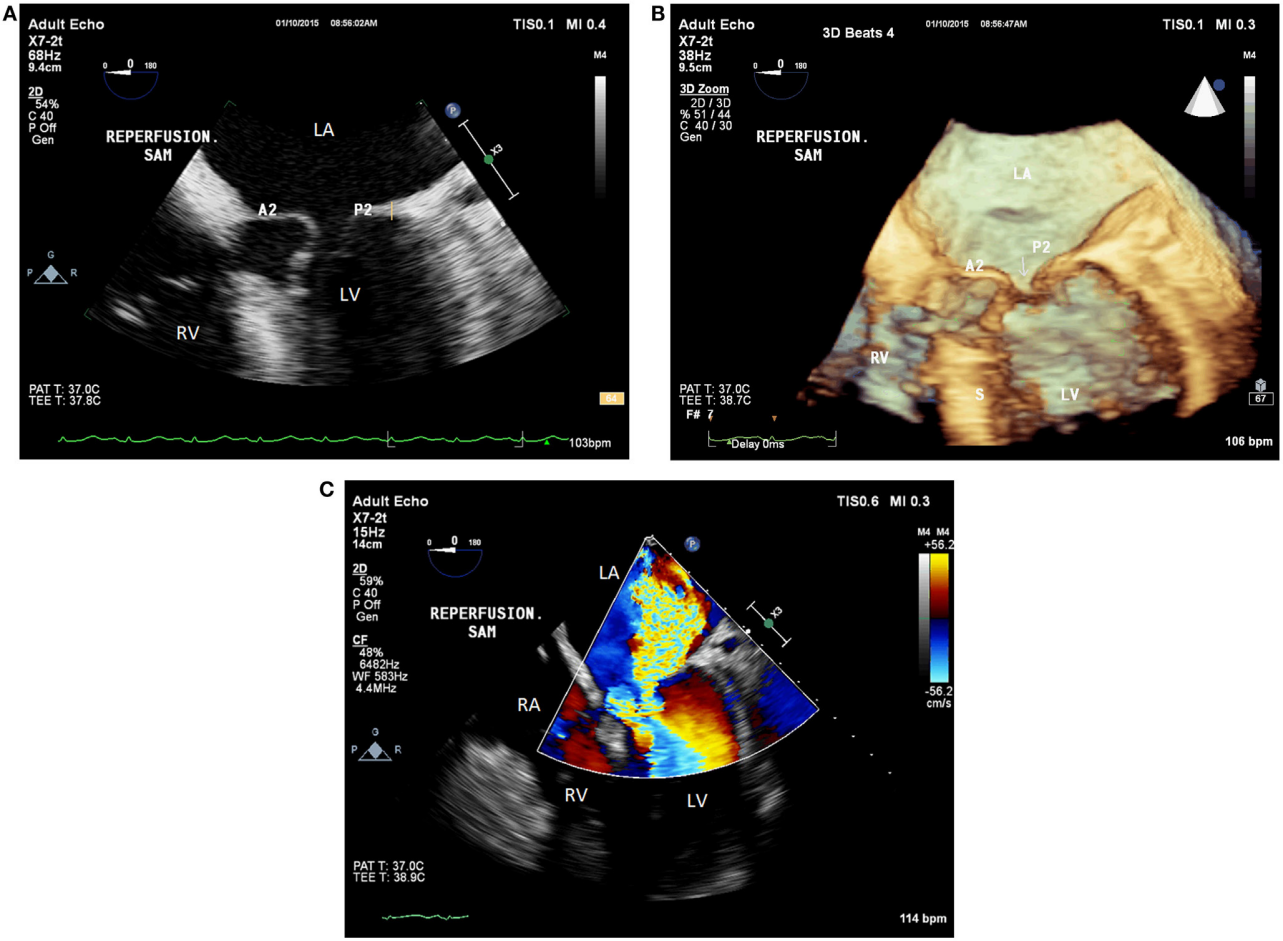
**(A)** Postreperfusion 2D TEE midesophageal modified five-chamber view demonstrating systolic anterior motion of the anterior mitral valve leaflet causing severe left ventricular outflow tract obstruction. **(B)** Postreperfusion 3D TEE midesophageal modified five-chamber view demonstrating left ventricular outflow tract obstruction resulting from systolic anterior motion of the anterior mitral valve leaflet. **(C)** Postreperfusion 2D TEE midesophageal modified five-chamber view with color flow Doppler, demonstrating severe posterolateral mitral regurgitation caused by systolic anterior motion of the anterior mitral valve leaflet. LV, left ventricle; RV, right ventricle; LA, left atrium; RA, right atrium; A2, second segment of the anterior mitral valve leaflet; P2, second segment of the posterior mitral valve leaflet; S, interventricular septum; Arrow, coaptation defect between the mitral leaflets.

Immediately after reperfusion, the patient became hypotensive (systolic BP in the 70s), tachycardic (HR of 105 beats/min), and hyperfibrinolytic likely due to PRS (12, 13). Continued hemodynamic support was given using 20 mcg of epinephrine, 500 mg of calcium chloride, 2 U of vasopressin, and aggressive volume resuscitation. This resulted in paradoxical hypotension (BP of 42/24 mmHg) and worsened tachycardia (HR of 114 beats/min). Transesophageal echocardiographic assessment demonstrated hyperdynamic LV function and an ejection fraction of 60–65%. It also revealed SAM of the anterior MV leaflet, causing DLVOTO and severe posterolaterally directed mitral regurgitation (Figures 2A–C). Based on the TEE findings, epinephrine and calcium chloride were discontinued. Boluses of esmolol and phenylephrine were administered. The patient was then placed in the Trendelenburg position and received a bolus of fluids. Manual compression of the abdominal aorta was also implemented to augment the SVR. This maneuver mimics aortic cross-clamp physiology and can help improve BP and coronary perfusion. The aortic compression was performed carefully to avoid compression of the inferior vena cava, which would have caused a further decrement in the preload. The treatment approach described above resulted in a rapid improvement of the patient’s hemodynamics, which included the following parameters: a BP of 127/65 mmHg, and a HR of 98 beats/min. An additional TEE examination indicated that both the SAM/DLVOTO and the mitral regurgitation had resolved. The remainder of the surgery was completed without any major hemodynamic derangements.

## Discussion

Liver reperfusion can be associated with significant hypotension resulting from a decrement in SVR and LV filling, an increment in PVR, myocardial depression, and bradyarrhythmias (1, 3–7, 14). This hemodynamic instability has predominantly been attributed to PRS, which has an occurrence rate of 12–81% during OLT (1–5, 9, 10, 14). The PRS is characterized as the combination of two factors occurring within 5 min of reperfusion of the portal vein: (1) hypotension lasting at least 1 min after reperfusion (defined as a mean arterial pressure reduction of 30% or greater) and (2) fibrinolysis (1–4, 9, 12). Different approaches have been utilized to prevent PRS, and multiple treatment options exist to prevent cardiovascular collapse. Preventative measures primarily consist of prophylactically administering fluids and vasoactive medications such as inotropes (e.g., epinephrine), vasopressors (e.g., phenylephrine), or calcium chloride, and flushing the allograft before reperfusion to eliminate vasoactive mediators and the preservative solution (1, 2, 9, 14, 15). However, other factors may be responsible for refractory hypotension after reperfusion, such as SAM and DLVOTO (6, 11). An increment in HR and contractility from the treatment of the PRS with inotropes, compounded by the vasodilatory state of reperfusion, and the relative hypovolemia (due to the allograft uptake of blood), may collectively precipitate the development of SAM/DLVOTO (6, 7, 16). If an expedient diagnosis of SAM/DLVOTO is not made, circulatory collapse may develop. Thus, a critical diagnostic dilemma exists for the transplant anesthesiologist. This is especially true if intraoperative TEE monitoring is not utilized.

Systolic anterior motion/DLVOTO is a common occurrence after MV repair with an incidence between 2 and 16% (17, 18). However, during liver transplantation, SAM/DLVOTO has been reported as the cause of hemodynamic compromise in a few cases where TEE was utilized for hemodynamic monitoring (6–8, 16, 19, 20). Maslow et al. characterized several preoperative echocardiographic parameters that can lead to anterior displacement of the MV coaptation point and cause DLVOTO in systole after MV repair (17). These risk factors include long MV leaflets, anterior papillary muscle displacement, a small ratio of the anterior to posterior MV leaflet coapted lengths (<1.3), a small LV basal systolic internal dimension (<4 cm), and a small distance between the MV coaptation point and the LV basal septum (<2.5 cm) (17). Other risk factors also exist, such as hypertrophic cardiomyopathy, LV basal septal hypertrophy, and an aorto-mitral angle reduction (<120°) (18). The presence of these risks factors, along with tachycardia, increased LV contractility and a reduction in preload and afterload, may ultimately result in SAM/DLVOTO after MV repair (17, 18). Unfortunately, due to the rarity of SAM in OLT patients, echocardiographic predictors for the development of SAM/DLVOTO during liver transplantation have not been clearly delineated. However, the presence of a long anterior MV leaflet and LV basal septal hyperpertrophy predisposes a patient to SAM during reperfusion, especially when the internal dimension of the LV is significantly reduced. Furthermore, the presence of a normal baseline TEE does not guarantee that a patient would not develop SAM/DLVOTO during liver allograft reperfusion secondary to the dynamic nature of the reperfusion period.

The patient described in this case report had a long anterior MV leaflet, and mild LV basal septal hypertrophy on his baseline intraoperative echocardiogram. After the onset of PRS, treatment was altered slightly to use calcium chloride, epinephrine, and vasopressin. Paradoxical hypotension surfaced as a result and necessitated another TEE examination. Based on the TEE findings, SAM/DLVOTO was rapidly diagnosed as the root cause, and the anesthesiology team was able to formulate a favorable treatment plan. This plan included discontinuation of inotropes, administration of esmolol and phenylephrine, augmentation of preload through Trendelenburg positioning and administration of fluids, and augmentation of SVR by manually compressing the abdominal aorta. Using these approaches rapidly improved the patient’s hemodynamics and resulted in a favorable outcome. The occurrence of tachycardia, increased LV contractility and relative hypovolemia, compounded by the long anterior MV leaflet and LV basal septal hypertrophy, was a perfect setup for SAM/DLVOTO after reperfusion (6–8, 16, 19). The inability to accurately predict the severity of PRS, and the effect of inotropic support using conventional invasive hemodynamic monitoring, makes TEE monitoring extremely beneficial. Without TEE monitoring, the timely diagnosis of SAM/DLVOTO would have been impossible. Thus, TEE complements standard invasive hemodynamic monitors during OLT.

In a previously published report, Aniskevich et al. described a similar case of SAM/DLVOTO after reperfusion. Their patient had concentric LVH and a normal preoperative dobutamine stress echocardiogram, with a maximal HR of 139 beats/min (6). The development of SAM/DLVOTO intraoperatively was likely caused by several factors: preexisting LVH (causing LV intracavitary hypovolemia), intraoperative use of dopamine, reperfusion vasodilatation/hypovolemia, hyperdynamic circulatory state, and epinephrine use (during reperfusion). A delayed diagnosis of SAM/DLVOTO was made after TEE examination, and therapy was changed from inotropes to labetalol. This resulted in a rapid improvement of the patient’s hemodynamics. Though similar, the unique aspect of our case was the rapid echocardiographic diagnosis of SAM/DLVOTO due to our institution’s policy to use TEE monitoring during OLT. Even though epinephrine is the drug of choice for PRS treatment in many centers, liver transplant physicians should be cautious of the potentially detrimental effect of inotropes on BP during reperfusion. Likewise, they should also utilize TEE whenever possible to help guide the management of refractory hypotension.

Transesophageal echocardiographic monitoring in OLT is extremely useful for hemodynamic monitoring due to its rapid diagnostic capabilities (21). Most notably, it can assist in the rapid diagnosis and subsequent treatment of the following conditions: SAM/DLVOTO, cardiovascular thrombus formation and pulmonary thromboembolism, valvular dysfunction, venous air embolism, paradoxical embolization (in the setting of an intracardiac or transpulmonary shunt), and acute right ventricular failure (6–8, 11, 16, 19–22). Both the *2003 ACC/AHA/ASE Guideline Update for the Clinical Application of Echocardiography* and the *2010 ASA/SCA Practice Guidelines for Perioperative TEE* strongly recommend the use of TEE in cases associated with significant hemodynamic swings, which includes OLT as a Class I recommendation (23, 24). Nonetheless, the benefits of TEE monitoring should always be weighed against its potential risks, especially in the setting of esophageal variceal disease. The *2010 ASA/SCA Practice Guidelines for Perioperative TEE* firmly emphasizes this point and recommends precautionary measures, such as ordering a gastroenterology consultation and using a smaller TEE probe to prevent significant variceal bleeding (23). Needless to say, the safety of TEE in OLT has been characterized in multiple studies. In a retrospective study, Suriani et al. reported a 2% complication rate while utilizing TEE in OLT patients (*n* = 100). Only two TEE-related complications were reported: bradycardia (*n* = 1) and variceal bleeding (*n* = 1). However, the patient with variceal bleeding had a history of esophageal varices and required sclerotherapy 4 years prior to transplantation. It is important to note that only three anesthesiologists performed TEE examinations in this study; two were cardiothoracic anesthesiologists with significant TEE experience; and one was a less experienced anesthesiologist under the supervision of a skilled echocardiographer. The low complication rate observed in this study most likely resulted from a limited number of manipulations occurring throughout image acquisition. Another study utilized TEE in OLT recipients with known esophageal varices (*n* = 287). This study only reported a 0.003% variceal bleeding rate (25).

Our institution currently uses TEE in most liver transplantation surgeries, based on preoperative EGD findings. Fellowship-trained cardiovascular anesthesiologists with adequate TEE experience perform both the placement of the probe and the TEE examination. Pre-anhepatic baseline TEE examinations are limited to the midesophageal views, which focus on valvular competency, intracardiac and transpulmonary shunts, and global biventricular function. The transgastric views are generally avoided due to the high incidence of gastropathy in patients with end-stage liver disease. The postreperfusion examination focuses primarily on global biventricular systolic function and filling, identifying intracardiac masses, intracardiac air, and valvular pathology, such as SAM.

Extensive preoperative cardiac evaluation is necessary for transplant recipients. This is primarily due to the high prevalence of coronary artery disease (2.5–27%), cardiomyopathy, and electrophysiological abnormalities in this patient population (26–31). As part of the preoperative evaluation, a resting echocardiogram is recommended for every liver recipient. This helps physicians to assess biventricular function (systolic and diastolic), ventricular size and thickness, valvular function, biatrial dimension, right ventricular systolic pressure, intracardiac structural abnormality, and intrapulmonary arteriovenous malformations (26, 27). Very poor outcomes have been reported for OLT patients with coronary artery disease (50% mortality rate and 80% morbidity rate) since the heart is unable to withstand the increased physiologic stress (28). Likewise, patients with risk factors for CAD, such as diabetes, hypertension, and NASH cirrhosis, should undergo stress testing to rule out myocardial ischemia (26, 27, 29, 30, 32).

A pharmacological stress test is preferred for assessing myocardial ischemia in OLT recipients, secondary to their poor exercise tolerance; however, the optimal pharmacological stress agent remains to be determined (26, 33). Vasodilator stressors, such as adenosine and dipyridamole, induce myocardial ischemia by decreasing oxygen supply in the stenotic coronary arteries (steal phenomenon), whereas dobutamine increases oxygen demand through its chronotropic and inotropic effects (29, 33–35). Myocardial ischemia can be detected by any of these pharmacological agents, although dobutamine has proven more effective at inducing myocardial ischemia in patients with low-grade coronary artery stenosis (29, 31, 34, 36, 37). The utility of the vasodilator perfusion stress test in OLT recipients has been questioned since the baseline vasodilatory state is heightened in these patients (26). Therefore, a dobutamine stress echocardiogram may provide the best overall evaluation for risk stratification in OLT candidates. As a preoperative tool, it can assess myocardial ischemia and prior myocardial injury, pulmonary hypertension, global biventricular function, and delayed intrapulmonary shunting (7, 27, 30, 32). It can also induce LVOT gradients in patients with significant LV basal septal hypertrophy, due to its ability to increase cardiac output while reducing SVR (7, 27, 32).

Our patient had a benign preoperative TTE examination, so an adenosine technetium-99m sestamibi stress SPECT myocardial perfusion imaging study was obtained to rule out coronary ischemia. In retrospect, a dobutamine stress echocardiogram would have been more ideal; however, it is unlikely that DLVOTO would have been induced. Instead, intraoperative TEE monitoring became an invaluable tool for successfully managing this patient.

## Conclusions

This case report highlights the importance of intraoperative TEE monitoring during OLT, and further emphasizes the utility of TEE in clinical decision-making. During the treatment of PRS with inotropes, paradoxical hypotension can result from SAM/DLVOTO and cause catastrophic outcomes if not promptly diagnosed. TEE monitoring can aid the rapid diagnosis and subsequent treatment of SAM/DLVOTO following the onset of PRS.

## Author Contributions

All authors listed, have made substantial, direct and intellectual contribution to the work, and approved it for publication.

## Conflict of Interest Statement

The authors declare that the research was conducted in the absence of any commercial or financial relationships that could be construed as a potential conflict of interest.
